# Training and Detraining Effects Following a Static Stretching Program on Medial Gastrocnemius Passive Properties

**DOI:** 10.3389/fphys.2021.656579

**Published:** 2021-04-01

**Authors:** Masatoshi Nakamura, Kaoru Yahata, Shigeru Sato, Ryosuke Kiyono, Riku Yoshida, Taizan Fukaya, João Pedro Nunes, Andreas Konrad

**Affiliations:** ^1^Institute for Human Movement and Medical Sciences, Niigata University of Health and Welfare, Niigata, Japan; ^2^Department of Physical Therapy, Niigata University of Health and Welfare, Niigata, Japan; ^3^Department of Rehabilitation, Kyoto Kujo Hospital, Kyoto, Japan; ^4^Metabolism, Nutrition and Exercise Laboratory, Physical Education and Sport Center, Londrina State University, Londrina, Brazil; ^5^Institute of Human Movement Science, Sport and Health, University of Graz, Graz, Austria

**Keywords:** muscle stiffness, range of motion, stretch tolerance, ultrasound, passive torque, flexibility

## Abstract

A stretching intervention program is performed to maintain and improve range of motion (ROM) in sports and rehabilitation settings. However, there is no consensus on the effects of stretching programs on muscle stiffness, likely due to short stretching durations used in each session. Therefore, a longer stretching exercise session may be required to decrease muscle stiffness in the long-term. Moreover, until now, the retention effect (detraining) of such an intervention program is not clear yet. The purpose of this study was to investigate the training (5-week) and detraining effects (5-week) of a high-volume stretching intervention on ankle dorsiflexion ROM (DF ROM) and medial gastrocnemius muscle stiffness. Fifteen males participated in this study and the plantarflexors of the dominant limb were evaluated. Static stretching intervention was performed using a stretching board for 1,800 s at 2 days per week for 5 weeks. DF ROM was assessed, and muscle stiffness was calculated from passive torque and muscle elongation during passive dorsiflexion test. The results showed significant changes in DF ROM and muscle stiffness after the stretching intervention program, but the values returned to baseline after the detraining period. Our results indicate that high-volume stretching intervention (3,600 s per week) may be beneficial for DF ROM and muscle stiffness, but the training effects are dismissed after a detraining period with the same duration of the intervention.

## Introduction

Joint flexibility, known as range of motion (ROM), and muscle stiffness could be contributors to movements of daily living and sports performance. Previous studies have pointed out that a poor ROM (Witvrouw et al., 2001; Backman and Danielson, 2011) and increased muscle stiffness (Watsford et al., 2010; Pickering Rodriguez et al., 2017) are risk factors for sports injuries. Studies have also shown that increased muscle stiffness associated with antagonist muscle contractions can inhibit joint movement and might result in higher energetic/metabolic costs (Ueno et al., 2018; Blazevich, 2019). Therefore, it could be important to improve ROM and decrease muscle stiffness in sports and rehabilitation settings.

Many previous studies showed that ROM and muscle stiffness were significantly improved immediately after static stretching intervention (Morse et al., 2008; Nakamura et al., 2011; Cè et al., 2015; Longo et al., 2017). On the other hand, while there is a vast literature indicating significant increases in ROM following the stretching intervention programs (Medeiros and Martini, 2018; Thomas et al., 2018; Brusco et al., 2019), for muscle stiffness, some studies have found significant decreases (Akagi and Takahashi, 2014; Blazevich et al., 2014; Ichihashi et al., 2016; Nakamura et al., 2017, 2020; Longo et al., 2021), others have found no changes (Konrad and Tilp, 2014a,b; Konrad et al., 2015). In light of the conflict literature, Freitas et al. (2018) conducted a meta-analysis to verify the effects of stretching intervention programs and concluded that *no statistical changes were observed for the muscle-tendon mechanical properties (including the muscle stiffness) after a chronic stretching intervention*. However, both the short duration of interventions and the low volumes of stretching performed per week may be responsible for not allowing sufficient stimuli for muscle adaptations. To the best of our knowledge, the study that implemented the longest stretching duration per week was conducted by Longo et al. (2021) in previous studies (Akagi and Takahashi, 2014; Blazevich et al., 2014; Ichihashi et al., 2016; Nakamura et al., 2017, 2020; Longo et al., 2021), in which subjects performed stretching for 2,250 s per week (45 s * 5 sets * 2 exercises * 5 times/week). Moreover, a high in-session volume (i.e., a high “continuous” time under tension) may be responsible for changes in muscle stiffness (Ryan et al., 2008; Nakamura et al., 2013). To date, based on the above observation, to what extent high stretching volumes can in fact induce improvements in muscle mechanical properties is still unclear.

Another interesting issue could be to explore to what extent there is any retention effect after a stretching program in sports and rehabilitation settings. According to literature, also concerning the effect of detraining, findings are controversial. Cipriani et al. (2012) observed large improvements in ROM following 4 weeks of training, but moderate reductions after the 4 weeks of detraining. Willy et al. (2001) reported that the increases in ROM after 6 weeks of stretching returned to baseline after a 4-week detraining period. On the other hand, Guissard and Duchateau (2004) showed that the increases in ROM and the reductions in muscle stiffness obtained after a 6-week high-frequency stretching program were sustained after a 4-week detraining period. Therefore, a deeper insight is necessary to address the controversy to whether a stretching program would prompt sustained effects on muscle stiffness during the long-term (training and detraining).

Therefore, this study aimed to investigate the effects of a stretching intervention on muscle stiffness using a longer stretching duration per week than in any other studies (3,600 s/week). Interestingly, two mechanisms have been proposed to explain the changes in ROM after a stretching intervention program: the mechanical theory, which is based on changes in the mechanical properties of the muscle-tendon unit, such as a decrease in muscle stiffness; and the sensory theory, which is related to changes in tolerated passive torque, also known as stretch tolerance (Weppler and Magnusson, 2010; Freitas et al., 2018). In order to clarify which mechanism was responsible for the increase in ROM after the stretching intervention program, we proposed an intervention with a stretch stimulus sufficient enough in duration to change both the muscle stiffness and the stretch tolerance. In addition, we investigated changes in ROM and muscle stiffness after a detraining period with the same duration as the stretching intervention period. We hypothesized that the increased ROM and decreased muscle stiffness would be sustained after the 5-week detraining period owing to the high weekly volume.

## Materials and Methods

### Experimental Design

In this study, the participants visited the laboratory >72 h prior to the baseline measurement for a familiarization session. The ankle dorsiflexion ROM (DF ROM), passive torque, and ultrasound measurements were determined before (PRE) and after (POST) the 5-week of static stretching (SS) program in both the dominant and non-dominant legs. In addition, all variables were measured after a 5-week detraining period (De-Tr). After PRE assessment, we defined the dominant side (the side with which the participant preferred to kick the ball) as the intervention side (SS side) and the non-dominant side as the control side (CON side) to minimize between-group variability due to personal factors such as exercise and activity patterns (Akagi and Takahashi, 2014). To control any immediate SS intervention effects, POST assessment was performed >72 h after the 10th SS session. During the De-Tr period (5 weeks following POST assessment), participants were instructed not to perform any stretching and resistance training programs.

### Participants

Fifteen males participated in this study (age, 21.5 ± 1.5 years; height, 170.6 ± 5.3 cm; and body weight, 63.3 ± 8.0 kg). Inclusion criteria were as follows: no regular resistance training within the past 6 months, no neuromuscular disease, and no history of orthopedic disease. None of the participants was competitive athletes or engaged in regular resistance training or stretching programs for the lower limbs. All participants were fully informed about the procedures and purpose of this study, and they provided written informed consent. This study was approved by the Ethics Committee of the Niigata University of Health and Welfare (#18305), Niigata, Japan.

### Dorsiflexion Range of Motion and Passive Torque Assessment

The participants sat on a dynamometer chair with a 0° knee angle (i.e., the anatomical position) and adjustable belts fixed over the trunk and pelvis (Biodex System 3.0, Biodex Medical Systems Inc., Shirley, NY, United States). The participants were reclined (70° hip angle; 0° full extension) to prevent tension at the back of the knee. The footplate of the dynamometer was passively and isokinetically dorsiflexed at a speed of 5°/second from the neutral anatomical position to the dorsiflexion angle just before subjects started to feel discomfort or pain (Akagi and Takahashi, 2013; Nakamura et al., 2020; Sato et al., 2020b). Before the passive dorsiflexion assessment, two cycles of passive dorsiflexion were performed to familiarize the subjects and prevent a conditioning effect of passive stretching on the muscle-tendon stiffness (Konrad and Tilp, 2014b; Hirata et al., 2017). In addition, we visually confirmed that there was no heal displacement during passive stretching by investigators.

After familiarization trials, the subjects stopped the dynamometer by activating a hand-held safety remote button when they started to feel discomfort or pain, and the angle just before this point was defined as DF ROM. The measurement was performed twice, and the average value was used for future analysis. In addition, passive torque at DF ROM was defined as the stretch tolerance (Weppler and Magnusson, 2010; Mizuno et al., 2013).

Throughout the passive dorsiflexion test, participants were requested to relax completely and not offer any voluntary contraction. We confirmed that there was no voluntary contraction of the medial gastrocnemius (MG) by monitoring muscle activity on the surface electromyography (FA-DL-720-140; 4Assist, Tokyo, Japan). Surface electrodes (Blue Sensor N, Ambu A/S, Ballerup, Denmark) were placed on the muscle belly of the MG. After the passive dorsiflexion assessment, muscle activity of the MG was measured during a 3 s maximal voluntary isometric contraction (MVIC), and we confirmed that all data were collected during a relaxed state, i.e., did not show muscle activity exceeding 5% of MVIC (Nakamura et al., 2011). The muscle activity was filtered using a band-pass filter of 10–1,000 Hz before being digitally stored (10 kHz sampling rate) on a personal computer for offline analysis. Analysis was performed using PowerLab 8/30 (AD Instruments, Colorado Springs, CO, United States) and LabChart 7 (AD Instruments), and root-mean-square (RMS, 50 ms window) values were calculated.

### Muscle Stiffness Assessment of Medial Gastrocnemius

A B-mode ultrasound imaging device (LOGIQ e V2; GE Healthcare Japan, Tokyo, Japan) and an 8-MHz linear array probe were used to assess elongation of the MG during the passive dorsiflexion test. We obtained the longitudinal ultrasound images of the MG, which were synchronized to the passive torque and joint angle outputs. The ultrasound probe was placed on the distal MG, near to the muscle-tendon junction (MTJ). The ultrasound probe was secured with a standard orthopedic stocking to prevent movement of the probe during the passive dorsiflexion test. In POST and De-Tr measurements, ultrasound images were obtained and compared to images acquired at PRE to assure the same position of measurement. An acoustically reflective marker was placed on the skin under the ultrasound probe proximal to the MTJ of MG to verify that the probe remained stable during the measurement (Morse et al., 2008; Nakamura et al., 2011). Ultrasound MTJ images were quantified using open-source digital measurement software (Image J, National Institutes of Health, Bethesda, MD, United States). MTJ displacement was defined as the distance between MTJ and the reflective marker.

The muscle force of the MG was estimated by multiplying the measured passive torque by the relative contribution of the physiological cross-sectional area (18%) of the MG within the plantarflexor muscles (Kubo et al., 2002; Konrad et al., 2017) and then dividing by the moment arm of the triceps surae muscle which was determined to be the length of the triceps surae muscles at neutral position (90 degrees) of the ankle (50 mm; Kubo and Ikebukuro, 2019). Passive muscle stiffness (N/mm) was calculated as the change in the passive torque from the neutral ankle position (0°) to DF ROM (smallest angle among PRE, POST, and De-Tr) and divided by the MTJ displacement.

### Static Stretching Programs

The SS program was performed only on the intervention side with a stretching board (Asahi stretching board, Asahi Corp., Gifu, Japan). Participants stood erect with one foot on the stretching board and the other foot on its edge and both arms against a wall in front of the body for balance (Akagi and Takahashi, 2013, 2014; Sato et al., 2020a; Yahata et al., 2021). Stretching intensity was defined as the greatest tolerated dorsiflexion angle on the stretching board. Participants who could tolerate >35° dorsiflexion, which was the maximal angle permitted by the stretching board, were instructed to maintain the stretching intensity by moving their body mass forward. All SS sessions were performed in the laboratory under the direct supervision of a research team. The SS intervention was comprised of six sets of 300 s of SS for a total of 1,800 s in each session. The SS program was performed 2 days per week for 5 weeks at 3–4 days intervals for a total of 10 sessions and 18,000 s of SS. This weekly volume is about 2.0–13.0 times greater to what has been tested in the literature (Freitas et al., 2018; Nunes et al., 2020).

### Test-Retest Reliability of Measurements

The test-retest reliability of the measurement for DF ROM, passive torque at DF ROM, and muscle stiffness was determined by a coefficient variation (CV) and the intraclass correlation coefficient (ICC) using seven healthy males (age, 20.8 ± 0.9 years; height, 168.9 ± 5.0 cm; and body weight, 61.3 ± 6.2 kg). The CVs of the measurements for DF ROM, passive torque at DF ROM, and muscle stiffness were 4.7 ± 5.2, 7.2 ± 6.3, and 3.6 ± 3.1%, respectively, and the ICCs of the measurements were 0.973, 0.868, and 0.881, respectively.

### Statistical Analyses

IBM SPSS Statistics version 24.0 (IBM Corp., Armonk, NY, United States) was used to conduct statistical analyses. Between-side differences in DF ROM, passive torque at DF ROM, and muscle stiffness at PRE assessment values were determined using paired *t*-tests. For all variables, a two-way repeated measure ANOVA was performed using the factors of time (PRE vs. POST vs. De-Tr) and side (SS side vs. CON side) to determine the interaction and main effects. As a *post hoc* test, we used the Bonferroni multiple comparison test to determine significant differences in time on each side. The Spearman’s rank correlation coefficients (*r*_s_) were computed to quantify the relationship between PRE and POST measurements of DF ROM, passive torque at DF ROM, and muscle stiffness on the SS side. Statistical significance was defined as *p* < 0.05 and the descriptive data were reported as means ± SD.

## Results

The results of all variables were presented in Table 1. The paired *t*-test showed that there were no significant differences in any variables between the SS and CON sides in PRE assessments. The repeated two-way ANOVA indicated significant interaction effects for DF ROM (*p* = 0.036, *F* = 3.75, *η*_p_^2^ = 0.211), passive torque at DF ROM (*p* = 0.036, *F* = 3.75, *η*_p_^2^ = 0.211), and muscle stiffness (*p* = 0.043, *F* = 3.52, *η*_p_^2^ = 0.201). On the SS side, the DF ROM and passive torque at DF ROM measurements at POST were significantly increased compared to PRE measurements (*p* < 0.05), whereas there were no significant differences between PRE and De-Tr or POST and De-Tr measurements. Similarly, on SS side, the muscle stiffness value at POST was significantly decreased from the PRE value, and there were no significant differences between PRE and De-Tr or POST and De-Tr values. There were no significant changes in any of the variables in CON side.

**Table 1:** The effect of the static stretching (SS) program on DF ROM, passive torque at DF ROM, and muscle stiffness of medial gastrocnemius (MG) during passive dorsiflexion test.

		PRE	POST	De-Tr	Interaction effect
DF ROM (°)	SS side	16.1 ± 4.5	21.8 ± 5.7^*^	18.9 ± 7.4	*F* = 3.75, *p* = 0.036
	CON side	16.4 ± 4.7	16.2 ± 4.5	17.3 ± 6.4	*η*_p_^2^ = 0.211
Passive torque at DF ROM (Nm)	SS side	23.5 ± 5.6	30.1 ± 10.5^*^	29.2 ± 10.7	*F* = 3.75, *p* = 0.036
	CON side	26.1 ± 8.6	25.7 ± 8.5	26.4 ± 6.0	*η*_p_^2^ = 0.211
Muscle stiffness (N/mm)	SS side	6.5 ± 3.8	3.7 ± 2.1^*^	6.7 ± 8.6	*F* = 3.52, *p* = 0.043
	CON side	6.5 ± 6.1	6.5 ± 4.4	6.0 ± 8.5	*η*_p_^2^ = 0.201

Interaction effect for the comparison between intervention side (SS side) and control side (CON side) based on a two-way repeated measure analysis of variance on the right column. Before static stretching intervention program (PRE); after static stretching intervention program (POST); after detraining periods (De-Tr). Data presented as mean ± SD.

* Significantly (*p* < 0.05) different form PRE value.

**Figure 1:**
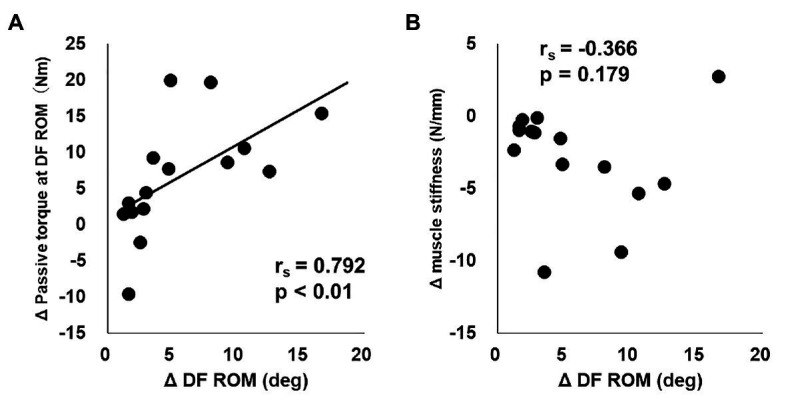
The relationships between change in dorsiflexion range of motion (DF ROM) and change in passive torque at DF ROM **(A)** or change in muscle stiffness **(B)** from before to after 5-week stretching intervention program in static stretching intervention side.

The relationships between changes in DF ROM, passive torque at DF ROM, and muscle stiffness were shown in Figure 1. Although a significant positive correlation was evident between the change in DF ROM and the change in passive torque at DF ROM (*r*_s_ = 0.792, *p* < 0.01; Figure 1A), there was no significant association between the change in DF ROM and the change in muscle stiffness (*r*_s_ = −0.366, *p* = 0.179; Figure 1B).

## Discussion

In this study, we investigated the effect of a 5-week high-volume stretching intervention program with 3,600 s of SS per week as well as the sustained effects after a 5-week detraining period. To the best of our knowledge, our study not only implemented the longest stretching duration per week of any study to date, but it was also the first to examine the detraining effect on passive muscle stiffness. Our results showed that after the 5-week intervention, DF ROM and passive torque at DF ROM were significantly increased, and muscle stiffness was significantly decreased. Contrary to our hypothesis, the increased DF ROM and decreased muscle stiffness returned to their baseline values after the 5-week detraining period.

Our results showing that DF ROM and passive torque at DF ROM increased after our 5-week stretching intervention program were consistent with findings in previous studies (Medeiros and Martini, 2018; Thomas et al., 2018; Brusco et al., 2019). However, while our results showing that the changes in DF ROM and passive torque at DF ROM returned to their baseline values after the 5-week detraining period were consistent with the study from Willy et al. (2001), they were not consistent with others, including Cipriani et al. (2012), who investigated the stretching intervention programs performed either daily or three times per week, and Guissard and Duchateau (2004), who investigated the stretching intervention programs performed five times per week. Our study, on the other hand, had a smaller SS frequency of two times per week. However, Cipriani et al. (2012) showed that there was no significant difference in changes in ROM between the two frequencies after the stretching intervention program and after the detraining period. However, it is unclear whether the training frequency influences the changes in ROM after the detraining period. Based on both previous studies and our results, it is possible that if the frequency of SS is less than twice a week, the improvements on ROM tend to be lost after a detraining period. Therefore, if a sustained effect is an objective, then an intervention frequency of three or more times per week may be necessary. Future research is required to determine whether the frequency of SS has an influence on sustaining ROM increases after the detraining period.

The results of this study also revealed that muscle stiffness was decreased after the 5-week high-volume stretching intervention program. While the meta-analysis conducted by Freitas et al. (2018) concluded that there was no significant decrease in muscle stiffness after a stretching intervention program, this was based on relative low SS volume interventions. Thus, we investigated a high-volume stretching intervention program of 3,600 s of stretching per week, and we determined that muscle stiffness was significantly decreased, suggesting that increasing the stretching time per week provided the muscles with the amount of stretching stimulus necessary for muscle adaptation and decreased muscle stiffness. Although the mechanism of muscle stiffness reduction was not determined in this study, recent reviews have stated that stretching programs alone do not significantly change fascicle length (Freitas et al., 2018; Nunes et al., 2020). Therefore, it is assumed that any change in fascicle length after our stretching intervention program was not a factor in the decreased muscle stiffness found in this study. Other factors, however, such as changes in the flexibility of the connective tissue surrounding the muscle fibers, are considered to have an influence on decreased muscle stiffness (Morse et al., 2008; Nakamura et al., 2012). Therefore, the decrease in muscle stiffness after our stretching intervention program could be associated with the change in the flexibility of the connective tissue.

Contrary to our hypothesis, the decrease in muscle stiffness after the stretching intervention program was returned to baseline value after a 5-week detraining period, which was consistent with the results of Guissard and Duchateau (2004), who found that decreased passive stiffness of the muscle-tendon unit following a 6-week stretching intervention program was not sustained after a 4-week detraining period. This discrepancy could be due to the difference in duration of the stretching intervention and detraining periods, or the difference in the weekly frequency of the stretching intervention. While the duration of the stretching intervention and the detraining periods were the same in our study, the duration of the detraining period was shorter than that of the intervention period in the comparative study (30 days vs. 42 days, respectively), and while the frequency of the stretching was only two times per week in our study, the frequency of stretching was five times per week in the study of Guissard and Duchateau (2004). This suggests that the stretching duration and frequency might influence the sustained effect of decreased muscle stiffness after a detraining period. Future studies are required to investigate the effect that different durations and frequencies have on sustaining decreased muscle stiffness after a detraining period.

As stated previously, the two mechanisms proposed to explain the changes in ROM after a stretching intervention program are the mechanical theory and the sensory theory (Weppler and Magnusson, 2010; Freitas et al., 2018). Our results showed a significant association between the change in DF ROM and the change in passive torque at DF ROM (*r*_s_ = 0.792, *p* < 0.01; Figure 1A), but no significant association between the change in DF ROM and the change in muscle stiffness (*r*_s_ = −0.366, *p* = 0.179; Figure 1B) after a 5-week stretching intervention program. These results correlate with previous studies (Kay et al., 2015; Fukaya et al., 2020; Kiyono et al., 2020), and suggest that the increase in DF ROM after a stretching intervention program is associated with a change in stretch tolerance, thus supporting the sensory theory.

Based on previous studies that have pointed out that poor ROM (Witvrouw et al., 2001; Backman and Danielson, 2011) and increased muscle stiffness (Watsford et al., 2010; Pickering Rodriguez et al., 2017) are risk factors for sports injuries and that muscle stiffness can inhibit joint movement of the antagonist muscle contraction and lower energetic/metabolic costs (Ueno et al., 2018; Blazevich, 2019), it can be expected that maintaining or improving ROM and decreasing muscle stiffness can lead to improvements in injury prevention, performance, and energy efficiency. However, these relationships are unclear in this study and warrant further research. Also, previous studies suggested that unilateral stretching intervention could affect ROM and muscle strength in contralateral side (Caldwell et al., 2019; Cè et al., 2020), so called cross-education effect. However, there were no significant changes in DF ROM and muscle stiffness in CON side (no-intervention side) in this study. Therefore, it is important to establish an effective protocol of occurring the cross-education effect after the stretching intervention program.

## Conclusion

Our study showed that DF ROM and passive torque at DF ROM were significantly increased after a 5-week high-volume stretching intervention program but returned to baseline after a 5-week detraining period. Similarly, our results showed that muscle stiffness was significantly decreased after the stretching intervention program, but also returned to baseline after a 5-week detraining period. Our results suggest that the increase in ROM after the stretching intervention program could be attributed to the change in passive torque at DF ROM, i.e., the stretch tolerance, which supports the sensory theory.

## Data Availability Statement

The raw data supporting the conclusions of this article will be made available by the authors, without undue reservation.

## Ethics Statement

The studies involving human participants were reviewed and approved by Ethics Committee of the Niigata University of Health and Welfare, Niigata, Japan. The patients/participants provided their written informed consent to participate in this study.

## Author Contributions

MN contributed to the study design and data collection and drafted and made the critical revisions to the manuscript. KY, SS, RK, and RY contributed to the data collection and made the critical revisions to the manuscript. TF, JN, and AK contributed to the study design and data analysis and made the critical revisions to the manuscript. All authors approved the final version of the manuscript and agreed to be accountable for all aspects of the work.

### Conflict of Interest

The authors declare that the research was conducted in the absence of any commercial or financial relationships that could be construed as a potential conflict of interest.
